# Breakdowns in Informativeness of Naturalistic Speech Production in Primary Progressive Aphasia

**DOI:** 10.3390/brainsci11020130

**Published:** 2021-01-20

**Authors:** Jeanne Gallée, Claire Cordella, Evelina Fedorenko, Daisy Hochberg, Alexandra Touroutoglou, Megan Quimby, Bradford C. Dickerson

**Affiliations:** 1 Program in Speech and Hearing Bioscience and Technology, Harvard University, Cambridge, MA 02138, USA; evelina9@mit.edu; 2 Frontotemporal Disorders Unit, Department of Neurology, Massachusetts General Hospital, Harvard Medical School, Boston, MA 02129, USA; ccordella@fas.harvard.edu (C.C.); dhochberg@mgh.harvard.edu (D.H.); atouroutoglou@mgh.harvard.edu (A.T.); mquimby@partners.org (M.Q.); 3 Department of Brain and Cognitive Sciences, Massachusetts Institute of Technology, Cambridge, MA 02139, USA

**Keywords:** primary progressive aphasia, informativeness, speech production

## Abstract

“Functional communication” refers to an individual’s ability to communicate effectively in his or her everyday environment, and thus is a paramount skill to monitor and target therapeutically in people with aphasia. However, traditional controlled-paradigm assessments commonly used in both research and clinical settings often fail to adequately capture this ability. In the current study, facets of functional communication were measured from picture-elicited speech samples from 70 individuals with mild primary progressive aphasia (PPA), including the three variants, and 31 age-matched controls. Building upon methods recently used by Berube et al. (2019), we measured the informativeness of speech by quantifying the content of each patient’s description that was relevant to a picture relative to the total amount of speech they produced. Importantly, form-based errors, such as mispronunciations of words, unusual word choices, or grammatical mistakes are not penalized in this approach. We found that the relative informativeness, or efficiency, of speech was preserved in non-fluent variant PPA patients as compared with controls, whereas the logopenic and semantic variant PPA patients produced significantly less informative output. Furthermore, reduced informativeness in the semantic variant is attributable to a lower production of content units and a propensity for self-referential tangents, whereas for the logopenic variant, a lower production of content units and relatively ”empty” speech and false starts contribute to this reduction. These findings demonstrate that functional communication impairment does not uniformly affect all the PPA variants and highlight the utility of naturalistic speech analysis for measuring the breakdown of functional communication in PPA.

## 1. Introduction

Primary progressive aphasia (PPA) is a clinical syndrome where aphasia is the initial predominant symptom due to neurodegenerative disease, most commonly frontotemporal lobar degeneration or Alzheimer’s disease [1]. The characteristics of aphasia in PPA are heterogeneous, and many patients present with a profile of language impairments that can be classified into one of the following three subtypes: the semantic variant (svPPA), the logopenic variant (lvPPA), or the non-fluent/agrammatic variant (nfvPPA) [2,3,4,5,6]. As distinct as these subtypes may be, they all share a devastating prognosis, i.e., as a patient’s aphasia progresses, his or her relationships will be adversely impacted by the breakdown of communication abilities [7,8,9].

A wide array of tests has been used to characterize language impairment in PPA [10,11] but concerns have been raised about highly constrained, decontextualized linguistic tasks being insufficient to describe or predict a person’s ability to communicate in everyday life, which is often referred to as “functional communication” [12,13,14,15]. One way to measure functional communication in patients with aphasia is through relatively naturalistic picture description tasks or structured interviews [9,14,16]. Despite concerns about the reliability of these methods [16], abnormalities of a variety of elements of speech and language in PPA can be successfully captured through the analysis of connected speech samples from such tasks. For example, the production of verbs and complex grammatical sentence structures is reduced in nfvPPA relative to lvPPA and svPPA [17,18], mirroring reports from more structured experimental tasks. To determine the extent to which communication is functional, researchers have measured breakdowns in the informativeness of speech production in patients with chronic aphasia by identifying words or phrases that are relevant to a picture or question, while ignoring form-based errors, such as mispronunciations of words, unusual word choices, and grammatical mistakes [16,19]. However, impairment in the informativeness of speech in PPA has received little investigation [20].

Berube et al. (2019) recently evaluated the informativeness of speech output in both stroke aphasia patients and PPA patients using a contemporary version of the Cookie Theft picture description task. They measured the patients’ production of words and phrases referring to concepts that were mentioned by control participants who described the same picture (i.e., “content units”) [21,22], and found that individuals with PPA and those with stroke aphasia conveyed less information about the picture than controls. These results provided further evidence that connected speech elicited in such a paradigm can be useful for quantifying functional communication abilities. However, the sample of PPA patients in Berube et al.’s study was small and did not allow for a detailed examination of potential between-variant differences.

We sought to investigate the breakdown of functional communication in PPA using a similar approach, aiming to answer two questions. First, as compared with controls, do patients in the mild stages of each of the three PPA variants exhibit reduced ability to convey information in a picture description task? Secondly, if multiple variants exhibit an impairment, do different factors contribute to the reduction in speech informativeness? In particular, we aimed to replicate the Berube et al. (2019) [21] method and compare it with our own proposed method of examining content production in naturalistic speech. Prior studies on svPPA have reported lower speech rate [17,23,24,25,26,27] but similar total numbers of words produced [18], with the important observation of fewer nouns and frequent semantic errors [17,18,23,24], in the presence of relative preservation of syntactic abilities [24,27]. Semantic variant PPA patients tend to produce words with higher frequency and less specificity [8,17,18,23,28]. With respect to lvPPA, which is perhaps the least well-understood variant, prior studies have observed lower speech rate [17,18,24], fewer open-class words [17,24], frequent phonemic errors [17,18,24], as well as numerous false starts and filled pauses [17,18]. As in svPPA, syntactic abilities appear to be largely preserved [18,24]. Finally, prior studies have reported that the speech of nfvPPA patients is slower [17,18,29,30,31] and contains fewer words [17,18,23,24,28], contains errors in closed-class words [25,26,32], syntactic agreement errors [26,28], few or no complex syntactic structures [26,28], and also exhibits lower narrative coherence [24,26,27].

These findings, combined with clinical observation, led us to formulate the following hypotheses: With regard to the first research question, we expected to find differences among the three variants in the patients’ ability to convey information relevant to the picture. The speech of svPPA patients was predicted to demonstrate reduced information content with each individual showing some degree of impairment; lvPPA patients’ information content was predicted to be reduced as a group but also to exhibit variability within the group, with some patients showing preserved informativeness of speech; finally, the nfvPPA group was predicted to show preserved informativeness, with few, if any, individuals demonstrating impairment. With regard to the second research question, svPPA patients were predicted to produce an abnormally high number of self-referential tangents and empty utterances in addition to reduced information content, whereas lvPPA patients were predicted to produce a high number of empty utterances and false starts in addition to reduced information content.

## 2. Materials and Methods

### 2.1. Participants

Data analyzed in this study were obtained from 101 participants, including 70 patients with a diagnosis of PPA and 31 age-matched controls. The PPA patients participated in this study as part of the Massachusetts General Hospital Frontotemporal Disorders Unit longitudinal PPA cohort study. Participants in this cohort underwent a comprehensive clinical evaluation as previously described [7,10]. The evaluation included a structured interview by a neurologist or psychiatrist covering cognition, mood/behavior, sensorimotor function, and daily activities; a neurologic examination, including office-based cognitive testing (for cases in this report, performed by B.C.D.); a speech-language assessment by a speech-language pathologist (for cases in this report, performed by C.C., M.Q., or D.H.), including the Progressive Aphasia Severity Scale (PASS) to specifically assess language impairment relative to a patient’s premorbid baseline [10]; and an MRI scan with T1- and T2-weighted sequences inspected visually by a neurologist. For all cases included here, visual inspection of the clinical MRI identified an atrophy pattern consistent with that typically seen in each PPA variant and ruled out other causes of focal brain damage [6].

For each participant, a clinician also performed a structured interview with an informant who knew the participant well (e.g., a spouse), augmented with standard questionnaires. For the participants in this report, the protocol included the National Alzheimer’s Coordinating Center (NACC) Uniform Data Set measures (using version 2.0 for 65 of the assessments, version 3.0 for the remaining 5), including Clinical Dementia Rating (CDR) scale supplementary language box ratings [10,33]. As part of the standard battery, connected speech samples were elicited through the Western Aphasia Battery–Revised (WAB-R) “Picnic Scene” task [34].

Individuals selected for this study had been diagnosed with imaging-supported svPPA, lvPPA, or nfvPPA according to consensus diagnostic criteria [3,6]. All participants and their care partners denied a pre-existing psychiatric disorder, other neurological disorder, or developmental cognitive disorder. Given the focus on mild PPA in the current study, we excluded participants with CDR language box scores above 1 (0 = normal language, 0.5 = very mild language impairment, 1 = mild language impairment, 2 = moderate language impairment, and 3 = severe language impairment). The PPA patient sample included 19 svPPA patients (mean age 69.6, SD 8.35, 11 females), 26 lvPPA patients (mean age 70.2, SD 7.17, 12 females), and 25 nfvPPA patients (mean age 68.2, SD 8.28, 15 females) (see Table 1 for further demographic information).

Data were also analyzed from thirty-one age-matched healthy controls, with no self-reported history of neurologic or psychiatric disorders, who participated in a longitudinal study conducted at the Speech and Feeding Disorders Laboratory at the MGH Institute of Health Professions (mean age 63.4, SD 8., 17 females). All participants in both samples were right-handed native English speakers. All participants (and their care partners for PPA patients) gave written informed consent in accordance with guidelines established by the Mass General Brigham Healthcare System Institutional Review Boards which govern human subjects research at Massachusetts General Hospital (protocol no. 2015P001363, protocol no. 2012P000432, protocol no. 2016P001421, protocol no. 2019P003391, protocol no. 2016P001594, and protocol no. 2013P001746). No significant between-group differences (svPPA, lvPPA, nfvPPA, and HC) were observed in sex or education level. The control group was observed to be younger than the lvPPA (*p* = 0.01) and nfvPPA (*p* = 0.004) groups. In the comparison of the PPA variants, no significant between-group differences (svPPA, lvPPA, and nfvPPA) were observed for the CDR language or PASS sum of boxes scores.

### 2.2. Speech Sample Collection

As part of their standard assessment, all PPA participants completed speech elicitation tasks including the WAB-R [24] Picnic Scene task; the connected speech samples obtained from this task were analyzed for the present study. Participants were presented the picture and prompted to provide a description of what they saw. Audio recordings were collected using a handheld Zoom H4N Recorder (Hauppauge, NY, USA) or an Olympus VN-702PC Voice Recorder (Center Valley, PA, USA) in a quiet room. For participants included in this analysis, the average speech sample durations were 73.7 s for svPPA (SD 28.0 s), 81.7 s (s) for lvPPA (SD 28.9 s), 104s for nfvPPA (SD 53.4 s), and 48.9 s for HC (SD 11.3 s).

### 2.3. Speech Sample Transcription and Basic Coding

Following data collection, speech samples were uploaded to a local hard drive on an encrypted device and preprocessed in Audacity^®^ Version 2.4.1 [35] to remove all clinician speech and background noise. Then, speech samples were transcribed and double-checked by two blinded (to diagnostic group) listeners. Inconsistencies in transcription were discussed and fixed upon consensus. Because the focus of the current study was on functional communication, phonemic errors (additions, omissions, and substitutions) where the intended word was clear were corrected during transcription (e.g., if the participant said, “they are sitting on a splanket” blanket was transcribed). Unrecognizable words were counted as empty words. Phonemic clusters followed by self-corrections or rerouting were counted as false starts. The average number of utterances (defined as any verbalization attempt, i.e., single words, including filler words, and false starts) was 135 for svPPA (SD 53.6), 135 for lvPPA (SD 60.3), 97.1 for nfvPPA (SD 34.1), and 150 for healthy controls (SD 65.9).

### 2.4. Content Unit (CUs) Coding

In order to analyze the informativeness of speech production, we followed the approach outlined by Berube et al. (2019) (see [36] for earlier work using this and related approaches). Using the healthy control samples, first, we created a corpus of 64 content units (CUs) (Table 2). A CU is defined as a unique concept. These concepts can correspond to an object/entity, an action, a property, or more abstract notions, such as spatial relations. A core motivation for the construct of CUs is to abstract over the potentially variable verbal descriptions that can be used for the same referent (e.g., “man”, “dad”, and “pops” all referring to a male individual). Following Berube et al. (2019) [21], the set of 64 CUs only included CUs that were each mentioned by at least three healthy controls. Then, we determined the number of CUs within each speech sample for all four groups. If a speech sample contained a CU more than once (even if the later occurrences used a different verbal description), only a single occurrence was counted. There were no penalties for the absence of any CUs.

**Figure 1:**
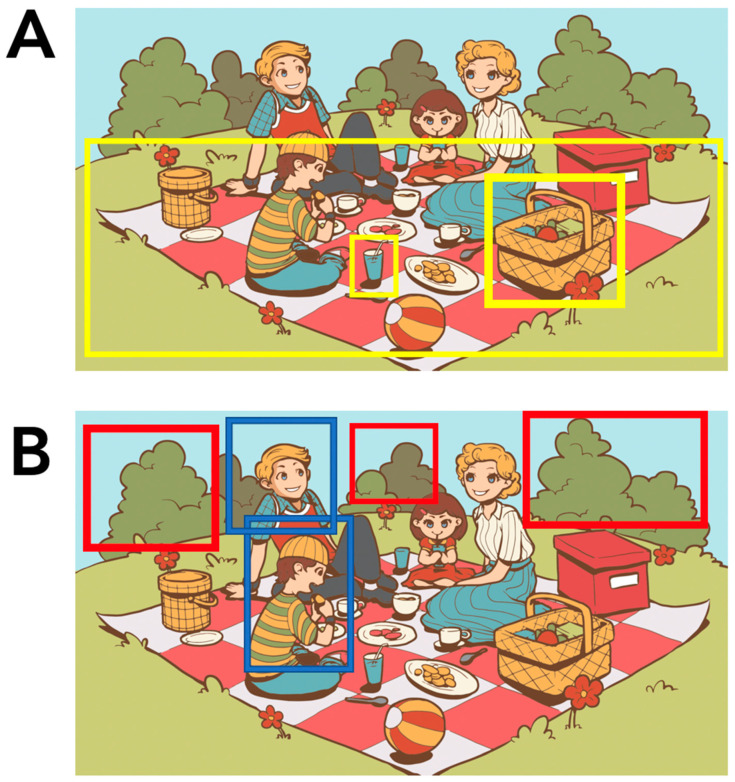
Identifying the range of ambiguity of elements in the Western Aphasia Battery-Revised (WAB-R) “Picnic Scene” task (Kertesz, 2007) [34]. In the study, the original WAB-R Picnic Scene was used, but due to permissions issues, here, we use a freely available picture to illustrate non-exhaustive examples of the range of ambiguity of possible referents in a picture description task. (**A**) The examples highlighted here (CU #24 “glass,” CU #25 “blanket”, CU #30 “basket”) are unambiguous entities similar to the ones found within the WAB-R Picnic Scene; (**B**) The examples highlighted here (CU #44 “shrubbery” (red) and CU #45 “fellow” (blue)) demonstrate the possible range of referents some CUs may represent. The image utilized for this example is freely available and reproduced with permission from Vecteezy (https://www.vecteezy.com/free-vector/picnic-basket).

In addition to Berube et al.’s (2019) [21] CU coding method, we developed an adjusted method to account for some ambiguity in the meaning of some CUs and compared the results of both methods. In particular, although the majority of CUs (42 of the 64, #1–42) unambiguously refer to a particular object/entity, action, or property of a specific object/entity in the picture (see Figure 1A for examples), the remaining 22 (#43–64) do not unambiguously refer to a particular aspect of the picture. Nine of these 22 CUs (#55–63) either refer to the scene overall or to concepts not physically present in the picture, inferred based on the schema of a picnic event (e.g., CU #63 “sandwich”, which is not actually present) or abstract in nature (e.g., CU #57 “music”). The remaining 13 CUs (#43–54, 64) are ambiguous with respect to which aspect(s) of the picture they refer to: in 2 CUs (#44, 54), the speech output does not help disambiguate the identity/scope of the referent (e.g., CU #44 “grass”/“yard”/“shrubbery”, and so forth), but in the remaining 12 CUs (#43, 45–53, 64), the speech output disambiguates toward one of two or more possible referents. For example, CU #45 where, e.g., “fellow” can refer to one of three entities (the man reading a book, the boy running with a kite, or the man fishing on the dock, see Figure 1B for examples), and based on the surrounding verbal context, it is possible to determine which of the referents the speaker is talking about. In an alternative CU coding scheme (CU-uniqueref, for ”unique referent”), for this set of 12 CUs we broke each CU down into further CUs based on the referent in question. In this coding scheme, if the same word is used again but now refers to a different object/entity, it is coded as a new CU. This alternative coding method could reveal biases in terms of which objects/entities patients tend to refer to, which may be different from the controls’ patterns and perhaps driven by the availability of different words.

For each of these two coding methods (the original Berube et al. (2019) [21] method and the CU-uniqueref method), we followed the original method by computing the total number of verbal descriptions that corresponded to CUs; thus, each participant was scored on the raw number of CUs. The counting of raw CUs in both the original method by Berube and colleagues (2019) [21] and our own CU-uniqueref method are non-normalized, and thus might be heavily influenced by sample length. This aspect of the CU coding process must be acknowledged as nfvPPA participants have been shown to produce less speech overall [17,18,23,24,28]. As such, between-group comparisons of the non-normalized measures of raw CU counts must be interpreted with caution and motivate a further, normalized analysis. For this reason, in contrast to the original Berube et al. (2019) [21] method, we also computed informativeness, i.e., raw CU count/all utterances, which places the raw CU count in the context of all the utterances produced by a given participant. Then, we used this normalized informativeness measure for subsequent interpretation of group differences.

### 2.5. Self-Referential, Empty, or Other Atypical Speech

**Figure 2:**
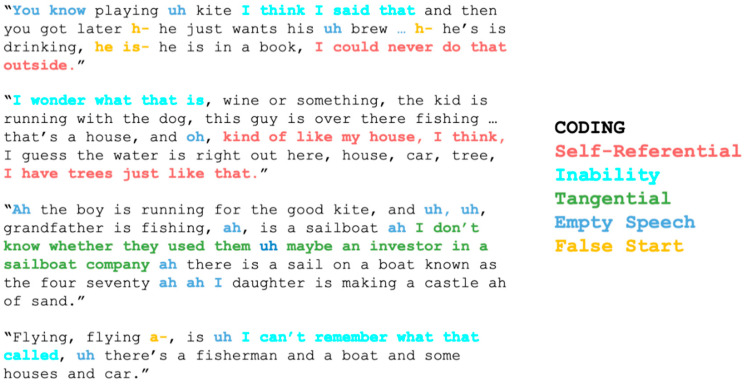
Examples of speech excerpts that were coded as self-referential, reflecting inability to decipher the picture or retrieve the right word, tangential, empty, or reflective of a false start.

To investigate the second research question regarding additional elements of speech samples that may contribute to reduced informativeness, such as self-referential or empty speech, we analyzed the speech samples for 5 additional types of output. A coder blinded to diagnostic group examined each sample for the presence of statements that (a) were self-referential (e.g., “I hate fish, I don’t eat fish, my husband eats salmon all the time” or “We have one of these”), (b) referred to inability to decipher the picture or retrieve the right word (e.g., “I’m not quite sure what that person is” or “Can’t think of that”), (c) were tangential to the contents of the picture (e.g., “Maybe an investor in a sailboat company” or “You don’t take a picnic right next to your house usually”), (d) were ”empty” speech (filler words or phrases, for example, e.g., “Uh no, yeah I guess”, “um, uh”), or (e) were false starts (unsuccessful lexical retrieval attempts and meaningless phonemic cluster productions). As with our measure of informativeness, the atypical speech analyses were also normalized in that they were calculated as a proportion for every participant. Controlling for speech sample length was an essential component in order to reduce bias in our subgroup comparisons. Inter-rater reliability was assessed for 20 of the 101 of the transcripts (five from each group), where the presence of a rating and rating type were judged for every single word per transcript and was found to be 97%. Further examples of these ratings can be seen in Figure 2.

### 2.6. Statistical Analysis

We descriptively compared the number of raw CUs and informativeness (CUs/all utterances) across methods and between groups using both the original Berube et al. (2019) [21] and the CU-uniqueref methods. Then, we conducted one-way ANOVAs to examine the main effect of group on the number of CUs produced and the informativeness of speech samples, followed up by post hoc t-tests and Hedges’ *g* effect sizes. Although length is a confound that must be considered in the interpretation of these ANOVAs, these analyses are still merited because there can be cases where even shorter sample lengths (as measured by total utterances) show relatively high numbers of CUs. Moreover, reduced sample length does not necessarily lead to reduced CU counts. While our measure of informativeness is normalized, we stress that our raw CU results are non-normalized and need to be interpreted with caution. To examine self-referential, empty, or other atypical speech, we calculated the proportion of words rated as belonging to one of five categories (self-referential, inability-related, tangential, empty speech, or false starts, see Section 2.5) relative to the total number of utterances. Then, we conducted one-way ANOVAs to examine the main effect of group on these 5 types of output. As in the prior analysis, post hoc *t*-tests and Hedge’s *g* effect sizes were calculated for every comparison. We set alpha at 0.05 and corrected for multiple comparisons as described below for each analysis. Statistical analysis was performed using R (Version 3.5.3 (2019-03-11)) [37].

## 3. Results

### 3.1. CUs and Informativeness

The complete sets of CUs generated for the original Berube et al. (2019) [21] and our CU-uniqueref methods are shown in Table 2. In the original Berube et al. (2019) method, we were able to generate 64 CUs. In the CU-uniqueref method, our specification of referents added 35 additional possible CUs to be scored.

For 49 out of 101 transcripts (9 svPPA, 8 lvPPA, 8 nfvPPA, and 24 HC), the total number of CU scores increased using the CU-uniqueref method relative to the Berube et al. (2019) method [21]. For 87.8% of score changes, the increase was one or two points (see Appendix B Table A2). Similarly, the informativeness scores of 49 out of 101 transcripts (9 svPPA, 8 lvPPA, 8 nfvPPA, and 24 HC) increased with the CU-uniqueref method. For 18.0% of score increases, the change in informativeness was greater than 1.5%.

There was a significant effect of group on the non-normalized raw CU production for both the original (F(3, 97) = 36.2, *p* < 0.001) and CU-uniqueref (F(3, 97) = 35.6, *p* < 0.001) methods (see Table 3). Post hoc comparisons were conducted using pairwise t-tests using Bonferroni adjusted alpha levels of 0.008 (0.05/6) and reported alongside Hedges’ *g* effect sizes. For both the original and CU-uniqueref methods, all three PPA variants demonstrated impaired production of unique CUs relative to HCs (*p* < 0.001 and *g* > 1.28). The nfvPPA participants produced fewer raw CUs than HCs, however, despite having the overall shorter speech samples relative to the other groups, raw CU count production was higher than that of the svPPA and lvPPA participants (*p*s < 0.005 and *g*s > 0.96, see Table 3). Across methods, there were no significant differences between lvPPA and svPPA.

**Figure 3:**
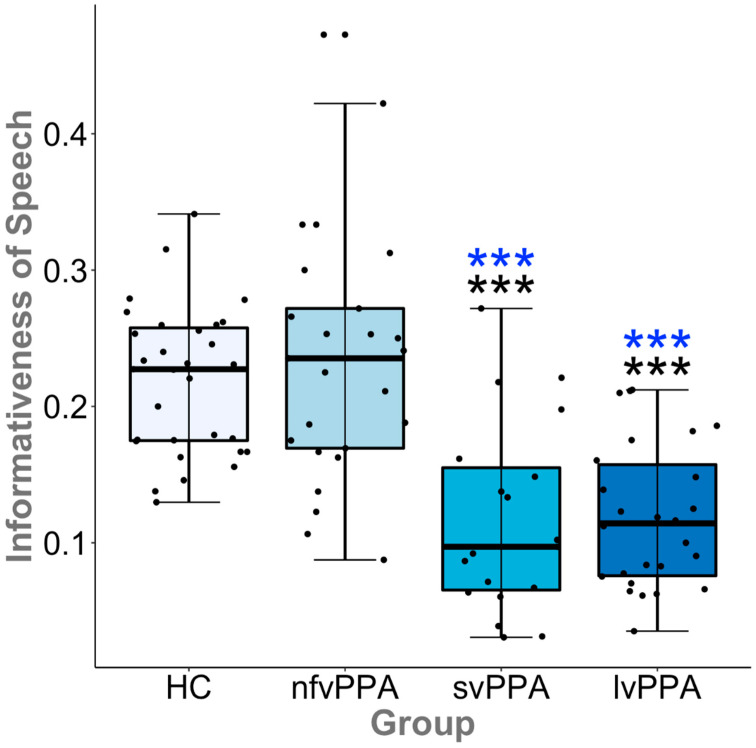
Illustrated here with the CU-uniqueref results, both the logopenic variant primary progressive aphasia (lvPPA) and semantic variant (svPPA) exhibited reduced informativeness of speech relative to controls, whereas the non-fluent/agrammatic variant (nfvPPA) group did not. The informativeness of speech output by the nfvPPA group was greater than that of lvPPA and svPPA. The error bars represent the 95% confidence interval of the median for each group. Significant differences are represented by asterisks (HC, black; nfvPPA, blue; *** *p* < 0.001).

Similarly, we found a significant effect of group on our normalized measure of informativeness (CUs/total utterances) for both the original (F(3, 97) = 20.6, *p* < 0.001) and CU-uniqueref (F(3, 97) = 21.1, *p* < 0.001) methods. As hypothesized, post hoc comparisons demonstrated that both svPPA and lvPPA exhibited impairment in the informativeness of speech relative to HCs (*p* < 0.001 and *g* > 1.27) across methods, whereas the informativeness of speech by the nfvPPA group was not impaired relative to HCs (see Figure 3). Across methods, the informativeness of nfvPPA output was greater than that of svPPA and lvPPA (*p*s < 0.001 and *g*s > 1.40), but there were no significant differences between svPPA and lvPPA. Contrary to one point in our first hypothesis, just as many individual lvPPA patients exhibited impaired informativeness as did svPPA patients (i.e., approximately eight or nine cases in each group fell within the 95% confidence interval for the HC distribution).

### 3.2. Self-Referential, Empty, or Other Atypical Speech

**Figure 4:**
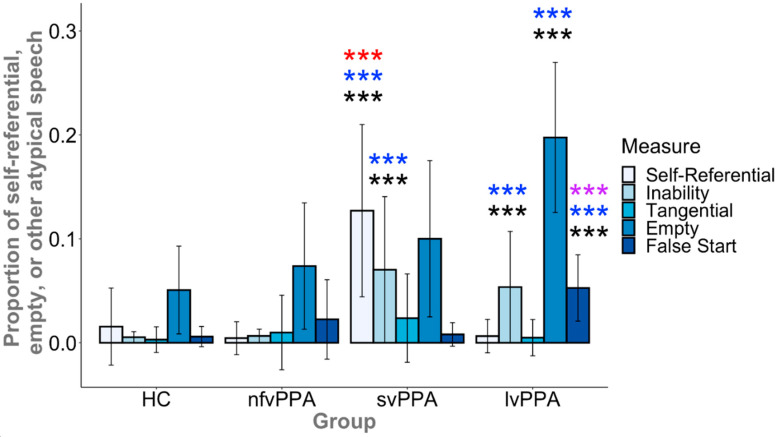
Relative to controls, the svPPA group exhibited more self-referential speech output and statements about inability, whereas the lvPPA group also exhibited more statements about inability, empty speech, and false starts. The nfvPPA group did not differ from HCs on any measure. The svPPA group exhibited more self-referential speech than lvPPA, whereas the lvPPA group exhibited more empty speech and false starts than svPPA. There were no significant group differences for tangential speech. The error bars represent one standard deviation from group means. Asterisks represent a significant difference relative to another group (HC, black; svPPA, purple; lvPPA, red; nfvPPA, blue; *** *p* < 0.001).

There was a significant main effect of group for the proportion of statements that were self-referential (F(3, 97) = 10.7, *p* < 0.001) or the statements in which the patient described their inability to do the task (F(3, 97) = 14.5, *p* < 0.001), as well as empty speech (F(3, 97) = 29.2, *p* < 0.001) and false starts (F(3, 97) = 17.9, *p* < 0.001) (see Figure 4 and Appendix C Table A3). A group effect for tangential statements was not present (F(3, 97) = 2.42, *p* = 0.071). Post hoc comparisons demonstrated that, as hypothesized, the speech samples of svPPA patients contained a greater proportion of self-referential statements than HC, lvPPA, and nfvPPA (*p*s < 0.001 and *g*s > 0.94). There were no significant differences among the HC, lvPPA, and nfvPPA groups (*p*s > 0.150). For statements about inability, both svPPA and lvPPA made more comments about their own difficulties with the task than nfvPPA (*p* < 0.001 and *g* > 1.09) and HC (*p*s < 0.001 and *g*s > 1.21), whereas nfvPPA and HC did not differ (*p* = 0.790). Whereas the overall effect of group only trended towards significance, svPPA produced numerically more tangential statements than HC (*p* = 0.058), however, there were no differences among the lvPPA, nfvPPA, and HC groups (*p*s > 0.536).

With regard to empty speech, as hypothesized, lvPPA produced significantly more empty output than HC (*p* < 0.001 and *g* > 1.29), svPPA and nfvPPA (*p*s < 0.001 and *g* > 1.01), whereas svPPA (*p* = 0.046) and nfvPPA did not differ from HC (*p* = 0.115). Finally, as hypothesized, lvPPA produced significantly more false starts than svPPA, nfvPPA, and HC (*p*s < 0.001 and *g*s > 0.84), however, there were no significant differences between svPPA and HC (*p* = 0.503), nfvPPA and HC (*p* = 0.043), nor svPPA and nfvPPA (*p* = 0.082).

## 4. Discussion

In this work, we asked two questions about functional communication in primary progressive aphasia (PPA). First, we asked whether patients in the mild stages of each of the three variants of PPA exhibit reduced ability to convey information in a picture description task. Second, we examined whether the increased production of types of speech output not directly relevant to the task may contribute to the reduction in speech informativeness. In particular, we asked whether patients with one of the three variants of PPA differ with respect to the production of self-referential, empty, or other atypical speech during this task. Such characteristics are often observed by clinicians and investigators, but they have not received sufficient attention in the prior literature.

To tackle the first question, we built on recent work by Berube et al. (2019) [21], who collected speech samples from individuals with aphasia using a picture description task and coded these for content units or CUs, i.e., concepts that are present in the output of healthy controls. Using this approach, Berube et al. (2019) [21] reported fewer CUs in the speech output of individuals with chronic stroke aphasia and of individuals with PPA. We proposed an adjustment to the coding method (which we termed CU-uniqueref) to disambiguate cases where the same CU could refer to one of multiple referents in the picture. Then, we compared the results of both of these methods. Furthermore, an additional unique contribution of our work was our normalized measure of informativeness, where we took participant-specific sample length into account. Given that raw CU counts do not account for semantically empty or irrelevant speech output, this measure more precisely targets functional communication ability as it reflects content efficiency. The reason this is important is that, for example, if a patient says five words or phrases that correspond to target concepts and nothing else, a listener could likely understand the patient’s point even if the patient’s speech is relatively sparse as compared with normal speech. In contrast, if another patient says five words or phrases that correspond to target concepts but also produces twenty words or phrases that include false starts, statements about how they cannot find the right word, and tangents, a listener would likely have greater difficulty understanding that person’s point. Raw CU count would be the same between the two, but the informativeness would be much lower in the second case.

We replicated Berube et al.’s finding that PPA patients produce fewer CUs than controls [21]. Critically, as predicted, we observed important differences among the three PPA variants in their ability to convey information relevant to the picture. In particular, svPPA and lvPPA patients’ speech demonstrated reduced informativeness, although the former group exhibited greater variability. The nfvPPA patients did not differ from controls in informativeness. These results suggest that grammatical impairment (a core feature of nfvPPA [6]) does not lead to a reduction in informativeness, whereas anomia (a core feature of both lvPPA and svPPA) and semantic memory impairment (a core feature of svPPA) likely do. Importantly, raw CU counts alone show reductions in all three variants relative to controls, consistent with prior work [17,18,19,20]; nonetheless, the magnitude of raw CUs produced by nfvPPA is still greater than for svPPA and lvPPA. Importantly, raw CU counts must be interpreted with caution as they do not reflect sample length. The risk of bias in the interpretation of raw CU counts motivated our measure of informativeness. Informativeness, the proportion of CUs to total utterances, demonstrates that the relative number of CUs that nfvPPA patients communicate is similar to that of healthy age-matched adults (i.e., nfvPPA patients do not produce more non-content related speech than controls, in contrast to the other two variants). While the content of nfvPPA speech samples were not diluted by atypical speech patterns (i.e., empty speech or false starts), the omission of closed-class words may have also contributed to preserved informativeness. This result speaks to the overall efficiency of nfvPPA speech relative to the other PPA variants. While previous work has reported upon the relative paucity of output in nfvPPA relative to the other variants [24,26,28], our results demonstrate that the amount of content relative to total output (i.e., informativeness) is preserved. This finding aligns with a recent systematic review [20], which concluded that word meanings and semantic structure appear to be largely preserved in this PPA variant. In our view, these findings demonstrate the value of examining both the raw volume of information, as well as the proportion of information in PPA. In line with prior reports of reduced informativeness [24,27,28,38], we found a reduction in informativeness in svPPA and lvPPA relative to controls and nfvPPA. These results are consistent with reports of reduced content word production in both of these variants [17,18,19,20].

To address the second question about additional factors that contribute to reduced informativeness of speech in svPPA and lvPPA, we quantified the presence of five types of task-irrelevant speech output, i.e., self-referential speech, speech referring to one’s inability to perform the task, tangential statements, empty speech, and false starts. As predicted, svPPA patients produced a large number of self-referential utterances about their own lives related to the scene depicted in the picture but tangential to the task, in addition to statements about their inability to decipher the picture, retrieve a word, or accurately describe a referent in the picture. Similar to svPPA, lvPPA patients produced many statements related to their inability to perform the task, but in contrast to svPPA, they did not produce self-referential statements, and instead had a large amount of empty (uninformative or indecipherable) speech (see Section 2.5 and Figure 2 for examples), as well as numerous false starts. The latter were plausibly related to failed lexical retrieval attempts. Thus, the reasons for reduced informativeness in these two variants are at least partially dissociable. As for nfvPPA, where the level of informativeness was similar to that of controls, the production of task-irrelevant speech was also similar to that of controls, both qualitatively and quantitatively.

A limitation of this work is that our naturalistic speech productions were prompted through a visual aid, rather than an open-ended prompt. As such, our findings speak to the informativeness of naturalistic speech within the constraints of a specific task. As such, we were unable to examine either the cohesion or pragmatics of discourse [20,37]. Another limitation is that the length of the speech samples varied by variant and by participant, where the shortest were produced by the nfvPPA group. However, as our primary measures of the proportion of content (informativeness) and atypical speech to total output were normalized, we consider this to be a minor issue in the interpretation of our results. Further consideration must be given to our methods of coding atypical speech. While our inter-rater reliability was quite high, only 20% of the transcriptions were cross-checked. This brings us to our final limitation, i.e., our methodology required extensive hand-coding and could not be automated. Thus, the replication or upscaling of our procedure with a larger sample size would be time-intensive for both the initial coding and reliability checks. However, given the irregularities of speech output in PPA, our hand-coding allowed for sensitivities to task-relevant speech, such as circumlocutions, and empty speech at the phrase-level, features that are unique to the individual.

In conclusion, the current results demonstrate that functional communication assessed in a task that closely approximates everyday interactions is not ubiquitously impaired across the PPA variants in the mild stages of the disease. Whereas both svPPA and lvPPA produce fewer CUs than controls, they each produce a larger amount of less meaningful speech, the types of which are partially dissociable, leading to an overall reduction in the informativeness of communication. In contrast, nfvPPA patients produce fewer CUs than controls but the speech they produce is informative. These findings highlight the value of assessing functional communication using paradigms that elicit naturalistic speech, and the utility of scoring the reductions in task-relevant speech output and also the increases in task-irrelevant speech output. Future directions of this work include a longitudinal analysis of informativeness in naturalistic speech production to monitor changes in the different variants as the disease progresses, and the potential application of this approach to evaluate outcomes of speech-language therapy.

## 5. Conclusions

Naturalistic speech samples can be used to identify differences between PPA variants and to shed light on the nature of language impairments. In the current study, we found that the informativeness of speech varies across groups, and critically, the nfvPPA group performed similarly to controls. Similarly, atypical patterns of speech vary across the PPA variants, where the nfvPPA group performs similarly to controls, whereas there are differences between lvPPA and svPPA.

## Figures and Tables

**Table 1:** Summary demographic information and clinical characteristics for the participants included in this study (see Appendix A Table A1 for participant-specific characteristics).

	PPA (*N* = 70)			Healthy Controls
	svPPA (*N* = 19)	lvPPA (*N* = 26)	nfvPPA (*N* = 25)	(HC) (*N* = 31)
Age at testing, years (SD)	69.7 (8.35)	70.2 (7.17)	68.2 (8.28)	63.4 (8.20)
Female, *n* (%)	11.0 (57.8)	12.0 (46.2)	15.0 (60.0)	17.0 (54.8)
Education, years (SD)	16.2 (2.30)	15.7 (2.38)	15.5 (2.73)	14.9 (1.83)
CDR language (SD), *n* = 0.5	0.71 (0.25), 11	0.71 (0.25), 15	0.66 (0.24), 17	--
PASS sum of boxes (SD)	3.50 (1.30)	4.04 (1.25)	3.82 (1.26)	--

**Table 2:** The complete set of 64 content units (CUs) identified in our healthy control sample (*n* = 31) based on Berube et al.’s (2019) methods [21]. The number of healthy control participants who produced a given CU in a speech sample is indicated in the “count” column. The unique referents for ambiguous CUs are represented in the “referent CU#” column, designating the agents, actions, and properties that at least three healthy control participants referred to by naming a particular CU.

Category	CU #	Referent CU#	Count	CU
Unambiguous entities/objects/events	**1**		31	Kite
	**2**		31	House, home, rental, cottage, cabin
	**3**		31	Wine, drink, liquid, drinks, beverage, soda, juice, champagne
	**4**		30	Dog, puppy
	**5**		29	Beach, sand
	**6**		28	Sailboat, boat
	**7**		28	Picnic, picnicking
	**8**		27	Woman, mother, mom, wife, lady
	**9**		26	Sandcastle(s), pile, castle
	**10**		26	Water, lake, ocean, river, sea
	**11**		26	Boy, son
	**12**		24	Girl, daughter
	**13**		21	Pier, dock
	**14**		18	Book
	**15**		18	Tree
	**16**		16	Car
	**17**		16	Driveway, path, road, street
	**18**		15	Garage, parked (car)
	**19**		15	Shoes, sandals, sneakers
	**20**		13	Fish (noun)
	**21**		13	Couple, two (people), parents
	**22**		13	Radio, boombox
	**23**		11	Flag, flagpole
	**24**		11	Glass, cup, glasses
	**25**		10	Blanket, carpet
	**26**		8	Shovel
	**27**		7	Pail, bucket
	**28**		7	Sky, clouds
	**29**		6	Fisherman
	**30**		4	Nasket
	**31**		3	(Man’s) glasses
	**32**		3	Bottle, thermos
Unambiguous actions	**33**		31	Fishing, caught (a fish), catching (a fish)
	**34**		31	Flying (a kite), pulling (a kite)
	**35**		31	Building (a sandcastle), making (a sandcastle), playing (in the sand), built a sandcastle
	**36**		26	Reading
	**37**		21	Pouring
	**38**		18	Enjoying, relaxing, happy, having a good time, relaxed, (having) fun
	**39**		17	(Dog is) chasing, (dog is) following, chased
	**40**		8	Sailing, cruising
Unambiguous properties	**41**		17	Beautiful, idyllic, nice, lovely, pleasant, calm
	**42**		10	Big, large
Ambiguous-referent entities	**43**		31	Man, father, dad, gentleman, grandpa, husband, hubby, pops
	43a	42	18	
	43b	29	15	
	**44**	No Pattern	31	Grass, yard, shrubbery, park, enclave, grassy, place, bushes, environment, foliage, gable, hill, mountains, scenery, spot, trees, forest
	**45**		30	Someone, guy, fellow, somebody, person, jabroni, adult
	45a	11		
	45b	12		
	45c	42		
	45d	29		
	**46**		24	Family, people, everyone, families, occupants
	46a	11, 12, 8, 42		
	46b	11, 12, 8, 42, 29		
	46c	No Pattern		
	**47**		12	Inlet, lakeside, seashore, seaside, bay, oceanside, shore, wharf
	47a	58		
	47b	12		
	47c	29		
	**48**		8	Kid, kids, children
	48a	11		
	48b	12		
	**49**		5	Shorts
	49a	11		
	49b	8 + 42		
	49c	42		
	**50**		3	T-shirt
	50a	11		
	50b	8		
Ambiguous-referent actions	**51**		12	Running
	51a	11		
	51b	4		
	51c	11 + 4		
	**52**		6	Sitting
	52a	8		
	52b	42		
	52c	8 + 42		
	**53**		5	Wearing
	53a	11		
	53b	8 + 42		
	53c	42		
	53d	29		
	**54**	15	4	Bloom, blossomed, blooming
Ambiguous-referent properties	**55**		27	Little, young, younger
	55a	11		
	55b	12		
	55c	4		
	55d	2		
	55e	43		
Unambiguous but inferred (not physically present/abstract)	**56**		20	Summer, sun, sunny, summertime, weather, season, spring, warm
	**57**		16	Music, listening to (music), (playing) music
	**58**		16	Day, afternoon, vacation, retreat
	**59**		9	Scene, picture
	**60**		9	Activities, recreational, sport
	**61**		8	Outside
	**62**		4	Breeze, windy
	**63**		4	Sandwich(es), food
Ambiguous-referent spatial relations	**64**		31	Across, around, background, behind, beside, close, distance, distant, far, foreground, front, left, nearby, next to, right, side
	64a	2		
	64b	6		
	64c	11		
	64d	15		
	64e	23		
	64f	4		
	64g	21		
	64h	29		
	64i	43		
	64j	16		
	64k	22		
	64l	17		

**Table 3:** The average CU and informativeness scores derived from both the original Berube et al. (2019) [21] and CU-uniqueref methods for all participants.

Method	Group	CUs (SD)	CU Effect Size vs. HCs (Hedge’s g)	Informativeness (SD)	Informativeness Effect Size vs. HCs (Hedge’s g)
Original Berube et al. (2019) [21]	HC	28.5 (6.46)	-	20.9% (5.67)	-
	lvPPA	14.5 (5.81) ***♦	2.25	11.7% (5.18) ***♦	1.75
	nfvPPA	20.3 (6.07) ***	1.28	23.1% (9.45)	−0.29
	svPPA	13.0 (5.67) ***♦	2.47	11.1% (6.46) ***♦	1.69
CU-uniqueref	HC	30.0 (7.99)	-	21.9% (5.87)	-
	lvPPA	14.8 (6.07) ***♦	2.23	11.9% (5.26) ***♦	1.86
	nfvPPA	20.8 (6.19) ***	1.33	23.5% (9.31)	>−0.22
	svPPA	13.7 (6.24) ***♦	2.34	11.7% (7.02) ***♦	1.65

*** *p* < 0.001 relative to HCs; ♦ *p* < 0.005 relative to nfvPPA.

## Data Availability

The data presented in this study are available on request from the corresponding author. The data are not publicly available as they contain information that could compromise the privacy of research participants.

## Appendix A

**Table A1:** Participant-specific demographic information.

Group	Participant	Sex	Age	Highest Educational Degree	CDR: Language	PASS Sum of Boxes
Semantic variant PPA	svPPA 1	F	68	Master’s	1	6
	svPPA 2	F	62	Bachelor’s	1	3
	svPPA 3	F	53	Bachelor’s	0.5	3
	svPPA 4	F	64	High School	0.5	3.5
	svPPA 5	M	54	Master’s	0.5	1.5
	svPPA 6	M	59	Bachelor’s	0.5	3
	svPPA 7	M	83	Bachelor’s	0.5	5
	svPPA 8	F	63	Bachelor’s	1	4.5
	svPPA 9	M	80	Bachelor’s	0.5	2
	svPPA 10	F	63	High School	0.5	4
	svPPA 11	F	70	Master’s	0.5	4.5
	svPPA 12	M	65	Bachelor’s	1	5
	svPPA 13	M	81	Bachelor’s	0.5	3
	svPPA 14	M	64	Doctorate	1	5
	svPPA 15	F	54	Doctorate	1	3
	svPPA 16	F	74	Bachelor’s	0.5	2.5
	svPPA 17	F	72	High School	0.5	4
	svPPA 18	F	65	Bachelor’s	1	3
	svPPA 19	M	68	Master’s	1	1
Logopenic variant PPA	lvPPA 1	M	68	Bachelor’s	1	5
	lvPPA 2	F	68	High School	0.5	3.5
	lvPPA 3	M	79	High School	0.5	3
	lvPPA 4	M	71	Master’s	0.5	0.5
	lvPPA 5	M	71	Master’s	0.5	3.5
	lvPPA 6	M	79	Master’s	1	3.5
	lvPPA 7	F	59	Bachelor’s	1	6
	lvPPA 8	F	70	Associate’s	1	5
	lvPPA 9	F	59	Master’s	1	6
	lvPPA 10	F	64	Bachelor’s	0.5	5
	lvPPA 11	M	71	Master’s	1	3.5
	lvPPA 12	M	72	High School	1	5
	lvPPA 13	F	53	Bachelor’s	0.5	4.5
	lvPPA 14	F	68	High School	1	3
	lvPPA 15	F	75	High School	0.5	5
	lvPPA 16	M	79	Bachelor’s	0.5	4
	lvPPA 17	F	70	Bachelor’s	0.5	3.5
	lvPPA 18	F	69	Bachelor’s	0.5	3
	lvPPA 19	M	75	Bachelor’s	1	5
	lvPPA 20	M	76	Master’s	0.5	3.5
	lvPPA 21	F	78	Master’s	0.5	5
	lvPPA 22	F	55	High School	0.5	5
	lvPPA 23	M	69	Doctorate	0.5	3
	lvPPA 24	M	72	Master’s	1	4
	lvPPA 25	M	73	Bachelor’s	1	5
	lvPPA 26	M	69	Bachelor’s	0.5	2
Non-fluent variant PPA	nfvPPA 1	F	72	Bachelor’s	0.5	3.5
	nfvPPA 2	M	64	Bachelor’s	0.5	5.5
	nfvPPA 3	F	69	Master’s	1	3
	nfvPPA 4	F	74	Bachelor’s	0.5	5
	nfvPPA 5	M	60	Bachelor’s	1	5
	nfvPPA 6	M	63	Bachelor’s	0.5	4
	nfvPPA 7	M	70	Doctorate	0.5	2
	nfvPPA 8	F	69	Master’s	1	3
	nfvPPA 9	F	63	High School	0.5	4
	nfvPPA 10	M	68	Bachelor’s	1	4
	nfvPPA 11	F	75	Doctorate	0.5	2
	nfvPPA 12	M	67	Bachelor’s	0.5	3.5
	nfvPPA 13	F	65	Bachelor’s	0.5	5.5
	nfvPPA 14	F	79	High School	0.5	5.5
	nfvPPA 15	M	55	Master’s	0.5	3
	nfvPPA 16	M	80	Master’s	0.5	3
	nfvPPA 17	F	71	Master’s	0.5	1
	nfvPPA 18	F	78	High School	0.5	3.5
	nfvPPA 19	M	69	Bachelor’s	1	5
	nfvPPA 20	F	75	High School	0.5	3.5
	nfvPPA 21	F	79	High School	0.5	5
	nfvPPA 22	F	82	High School	1	6
	nfvPPA 23	M	77	High School	0.5	3
	nfvPPA 24	F	74	High School	1	4
	nfvPPA 25	F	62	Master’s	1	3
Healthy Controls	HC 1	F	61	Bachelor’s		
	HC 2	F	61	High School		
	HC 3	M	53	Bachelor’s		
	HC 4	F	50	High School		
	HC 5	M	52	Bachelor’s		
	HC 6	M	51	Bachelor’s		
	HC 7	M	68	Bachelor’s		
	HC 8	F	71	Bachelor’s		
	HC 9	F	71	Bachelor’s		
	HC 10	M	62	High School		
	HC 11	F	71	Bachelor’s		
	HC 12	F	68	Bachelor’s		
	HC 13	M	69	Bachelor’s		
	HC 14	M	53	Bachelor’s		
	HC 15	F	55	Bachelor’s		
	HC 16	M	68	Bachelor’s		
	HC 17	F	66	High School		
	HC 18	M	65	Bachelor’s		
	HC 19	F	62	Bachelor’s		
	HC 20	F	66	Bachelor’s		
	HC 21	M	59	Bachelor’s		
	HC 22	F	57	High School		
	HC 23	M	54	Bachelor’s		
	HC 24	F	60	Bachelor’s		
	HC 25	F	72	High School		
	HC 26	M	73	Bachelor’s		
	HC 27	F	55	High School		
	HC 28	F	65	Bachelor’s		
	HC 29	F	63	Bachelor’s		
	HC 30	M	76	High School		
	HC 31	M	83	Bachelor’s		

## Appendix B

**Table A2:** The number of participants whose score increased by 1-4 points with the CU-uniqueref method.

Group	0 pt.	1 pt.	2 pt.	3 pt.	4 pt.	No. Participants Changed
HC	7	9	11	2	2	24
lvPPA	18	7	0	1	0	8
nfvPPA	17	4	4	0	0	8
svPPA	10	6	2	1	0	9

## Appendix C

**Table A3:** The proportion of self-referential and other off-topic or empty speech.

Group	Self-Referential Statements	SD	Ability Statements	SD	Tangential Statements	SD	Empty Speech	SD	False Starts	SD
HC	0.016	0.037	0.005	0.013	0.003	0.012	0.051	0.042	0.006	0.009
lvPPA	0.006	0.016	0.053	0.056	0.005	0.017	0.198	0.072	0.053	0.032
nfvPPA	0.004	0.016	0.007	0.019	0.009	0.036	0.074	0.061	0.023	0.038
svPPA	0.127	0.183	0.070	0.067	0.024	0.043	0.100	0.075	0.008	0.011
